# Curdlan, a Microbial β-Glucan, Has Contrasting Effects on Autoimmune and Viral Models of Multiple Sclerosis

**DOI:** 10.3389/fcimb.2022.805302

**Published:** 2022-02-07

**Authors:** Fumitaka Sato, Yumina Nakamura, Aoshi Katsuki, Sundar Khadka, Ijaz Ahmad, Seiichi Omura, Nicholas E. Martinez, Ikuo Tsunoda

**Affiliations:** ^1^ Department of Microbiology, Kindai University Faculty of Medicine, Osakasayama, Osaka, Japan; ^2^ Department of Microbiology and Immunology, Center for Molecular and Tumor Virology, Louisiana State University Health-Shreveport, Shreveport, LA, United States

**Keywords:** animal models, CNS demyelinating diseases, neuropathology, persistent virus infections, *Picornaviridae* infections, fungal infections, principal component analysis, bioinformatics

## Abstract

Multiple sclerosis (MS) is an immune-mediated disease characterized by inflammatory demyelination and axonal degeneration in the central nervous system (CNS). Bacterial and fungal infections have been associated with the development of MS; microbial components that are present in several microbes could contribute to MS pathogenesis. Among such components, curdlan is a microbial 1,3-β-glucan that can stimulate dendritic cells, and enhances T helper (Th) 17 responses. We determined whether curdlan administration could affect two animal models for MS: an autoimmune model, experimental autoimmune encephalomyelitis (EAE), and a viral model, Theiler’s murine encephalomyelitis virus (TMEV)-induced demyelinating disease (TMEV-IDD). We induced relapsing-remitting EAE by sensitizing SJL/J mice with the myelin proteolipid protein (PLP)_139-151_ peptide and found that curdlan treatment prior to PLP sensitization converted the clinical course of EAE into hyperacute EAE, in which the mice developed a progressive motor paralysis and died within 2 weeks. Curdlan-treated EAE mice had massive infiltration of T cells and neutrophils in the CNS with higher levels of Th17 and Th1 responses, compared with the control EAE mice. On the other hand, in TMEV-IDD, we found that curdlan treatment reduced the clinical scores and axonal degeneration without changes in inflammation or viral persistence in the CNS. In summary, although curdlan administration exacerbated the autoimmune MS model by enhancing inflammatory demyelination, it suppressed the viral MS model with reduced axonal degeneration. Therefore, microbial infections may play contrasting roles in MS depending on its etiology: autoimmunity versus viral infection.

## Introduction

Multiple sclerosis (MS) is an immune-mediated disease characterized by inflammatory demyelination and axonal degeneration in the central nervous system (CNS) (Trapp and Nave, 2008). Although the etiology of MS is unclear, there are two major effector mechanisms that have been proposed as a cause/trigger of MS: autoimmune responses against myelin sheaths (autoimmune theory) and CNS viral infections (viral theory) (Sato et al., 2018). The autoimmune theory has been supported by clinical findings. For example, some MS patients have higher immune responses to CNS antigens than the healthy controls; immune cell infiltration and antibody deposition have been observed in demyelinating lesions (Lucchinetti et al., 2000). Immunomodulatory drugs, such as interferon (IFN)-β and anti-very late antigen (VLA)-4 antibody, have therapeutic efficacy in MS patients (Minagar et al., 2003). Experimentally, experimental autoimmune encephalomyelitis (EAE) has been widely used as an autoimmune model of MS, since EAE mimics human MS, clinically, histologically, and immunologically (Sato et al., 2016). EAE can be induced by sensitization with CNS antigens including myelin proteolipid protein (PLP), myelin oligodendrocyte glycoprotein (MOG), and myelin basic protein (MBP) (Miyamoto et al., 2006). In most EAE models, demyelination is mediated by pro-inflammatory myelin-specific CD4^+^ T helper (Th) 1 and Th17 cells; axonal degeneration occurs secondary following severe demyelination (Tsunoda and Fujinami, 2002).

The viral theory of MS has also been supported clinically and experimentally (Libbey et al., 2014). For example, some viruses including human herpesvirus 6 have been isolated from the serum and brain samples of MS patients; increased anti-viral immune responses have also been detected in cerebrospinal fluid of MS patients (Omura et al., 2018). Experimental infections of neurotropic viruses, including Theiler’s murine encephalomyelitis virus (TMEV) and mouse hepatitis virus, can induce MS-like CNS diseases in animals (Trandem et al., 2010; Kulcsar et al., 2014). TMEV is a non-enveloped, positive-sense, single-stranded RNA virus that belongs to the family *Picornaviridae* (Hertzler et al., 2000) and has been widely used as a viral model of MS. TMEV-infected mice develop chronic inflammatory demyelination and axonal degeneration in the CNS with viral persistence in macrophages and glial cells, including myelin-forming oligodendrocytes (Sato et al., 2017). TMEV-induced demyelinating disease (TMEV-IDD) has two components in the pathogenesis: 1) direct lytic viral infection (viral pathology) and 2) immunopathology, in which CD4^+^ and CD8^+^ T cells, antibody, and macrophages have been demonstrated to play pathogenic roles. In TMEV-IDD, axonal degeneration has been shown to precede demyelination (Tsunoda et al., 2003).

In addition to viral infections, bacterial and fungal infections have been associated with MS, since the higher levels of antibody responses to certain bacteria and fungi, such as *Chlamydia pneumoniae* and *Candida albicans*, have been detected in some MS patients than in the healthy controls (Alonso et al., 2018; Cossu et al., 2018). Experimentally, in the MOG_35-55_-induced EAE, injection of *Streptococcus pneumoniae* during the latent stage exacerbated the clinical signs (Herrmann et al., 2006). *C. albicans*-injected mice prior to MOG sensitization also developed more severe EAE than the control mice (Fraga-Silva et al., 2015). Despite the previous reports supporting the role of bacterial and fungal infections, no single microbe has been identified to cause/trigger MS consistently. This raises the hypothesis that, instead of one single microbes, microbial components commonly present in several bacteria and fungi may contribute to MS pathogenesis.

One of such candidate microbial components is curdlan, one of the β-glucans that are components of a variety of bacteria/fungi, which are not synthesized in humans; β-glucans can be detected in human sera during fungal infections, as shown in a case report of MS (Pisa et al., 2011). Curdlan is a high molecular weight microbial 1,3-β-glucan with linear polymer of 1,3-β-glycosidic linkages; curdlan is recognized by the C-type lectin receptor dectin-1, a pattern recognition receptor, expressed on various myeloid cells including dendritic cells (DCs), monocytes, macrophages, B cells, and neutrophils (LeibundGut-Landmann et al., 2007; Isnard et al., 2021). Following curdlan stimulation, DCs enhance interleukin (IL)-17-producing Th17 cell responses. Although Th17 cells play a beneficial role in host defense against bacteria and fungi, such as *Klebsiella pneumoniae* and *C. albicans* (Curtis and Way, 2009), pro-inflammatory Th17 cells could play an effector role in inflammatory demyelination of MS (Hussman et al., 2016), EAE (Hofstetter et al., 2005; Komiyama et al., 2006), and TMEV-IDD (Hou et al., 2009; Martinez et al., 2015). Curdlan has also been reported to enhance IL-10 production and favor IgA antibody responses (LeibundGut-Landmann et al., 2007; Kawashima et al., 2012; Fujimoto et al., 2019). In addition to acquired immunity, β-glucans can induce a long-lasting change in innate immune cells (i.e., monocytes, macrophages, and microglia) (Haley et al., 2019), as a result of complex regulation including epigenetic reprogramming; the phenomenon is referred to as “trained innate immunity” or “innate immunity memory” (Rusek et al., 2018). The trained immunity independent of T or B cells has been shown to be beneficial in various microbial infections including bacteria, fungi, and viruses by diverse mechanisms, such as changes in cytokine and nitric oxide production; the anti-microbial protection can last for several weeks (Quintin et al., 2012). On the other hand, innate immunity stimulated by β-glucans could be deleterious, exacerbating immune-mediated diseases (Quintin, 2019).

In the current study, we investigated whether curdlan, a component present in many microbes, could affect autoimmune and viral models for MS, EAE and TMEV-IDD. We hypothesized that immune modulation by curdlan may result in different outcomes in the two MS models, whose immune effectors and neuropathology have been shown to be different. We first determined the effects of curdlan in EAE, where we injected mice with curdlan prior to sensitization with the PLP_139-151_ peptide. We found that curdlan-treated EAE mice developed a fatal hyperacute EAE, in which mice had progressive motor paralysis with massive infiltration of T cells and neutrophils in the CNS, although the control EAE mice developed relapsing-remitting (RR) EAE with mild to moderate CNS inflammation. We also found sustained higher levels of Th17 and Th1 responses in curdlan-treated EAE mice, compared with the control EAE mice. In contrast, curdlan treatment ameliorated TMEV-IDD with less severe clinical signs and lower levels of axonal degeneration in the CNS, compared with the control TMEV-infected group, although curdlan treatment did not alter inflammatory demyelination or viral persistence in the CNS. Thus, our findings suggested that microbial infections could result in different outcomes in MS depending on its etiology: autoimmunity versus viral infection.

## Materials and Methods

### Animal Experiments

We purchased 5-week-old SJL/J mice (JAX^®^ Mice Strain) from Charles River Laboratories Japan, Inc. (Yokohama, Japan) and maintained the mice under specific pathogen-free conditions in the animal care facility at Kindai University Faculty of Medicine (Osaka, Japan) or Louisiana State University Health-Shreveport (LSUHS, Shreveport, LA). All experimental procedures were reviewed and approved by the Institutional Animal Care and Use Committee of Kindai University Faculty of Medicine or LSUHS and performed according to the criteria outlined by the National Institutes of Health (NIH).

For EAE induction, we sensitized 6 to 7-week-old SJL/J mice subcutaneously (s.c.) with 100 nmol of the PLP_139-151_ peptide (United BioSystems Inc., Herndon, VA) emulsified in complete Freund’s adjuvant (CFA) composed of incomplete Freund’s adjuvant [Becton, Dickinson and Company (BD), Tokyo, Japan] and *Mycobacterium tuberculosis* H37 Ra (BD) (Tsunoda et al., 2007). The final concentration of *M. tuberculosis* in the PLP_139-151_/CFA emulsion was 2 mg/mL (400 μg/mouse). Clinical scores of EAE were evaluated as follows: 0, no signs; 1, paralyzed tail; 2, mild hind limb paresis; 3, moderate hind limb paralysis; 4, complete hind limb paraplegia; and 5, moribund or death (Omura et al., 2019).

For TMEV-IDD induction, we infected 6 to 7-week-old SJL/J mice intracerebrally (i.c.) with 2 × 10^5^ plaque forming units (PFUs) of the Daniels (DA) strain of TMEV (Kawai et al., 2015). Clinical signs of TMEV-IDD were evaluated by measuring impairment of righting reflex: the proximal end of the mouse’s tail was grasped and twisted to the right and then to the left (0, a healthy mouse resists being turned over; 1, the mouse is flipped onto its back but immediately rights itself on one side; 1.5, the mouse is flipped onto its back but immediately rights itself on both sides; 2, the mouse rights itself in 1 to 5 seconds; 3, righting takes more than 5 seconds; and 4, the mouse cannot right itself) (Tsunoda et al., 2008).

In both EAE and TMEV-IDD models, mice were injected intraperitoneally (i.p.) with 5 mg of curdlan (FUJIFILM Wako Pure Chemical Corporation, Osaka, Japan) in 200 μL of phosphate-buffered saline (PBS) one day prior to PLP sensitization or TMEV infection. Mice in the control group had PBS injection or no treatment.

### Neuropathology

We perfused EAE or TMEV-infected mice with PBS followed by a 4% paraformaldehyde (FUJIFILM Wako Pure Chemical Corporation) solution in PBS, harvested the spinal cord and brain, which were divided into 10 to 14 transversal segments and five coronal slabs, respectively, and embedded in paraffin. We stained 4-μm-thick sections with Luxol fast blue (Solvent blue 38; MP Biomedicals, LLC, Irvine, CA) for myelin visualization and performed histological scoring of the CNS, as described previously (Sato et al., 2013). For scoring of spinal cord sections, each spinal cord section was divided into four quadrants: the ventral funiculus, the dorsal funiculus, and each lateral funiculus. Any quadrant containing meningitis, perivascular cuffing (inflammation), or demyelination was given a score of 1 in that pathological class. The total number of positive quadrants for each pathological class was determined and then divided by the total number of quadrants present on the slide and multiplied by 100 to give the percent involvement for each pathological class. An overall pathology score was also determined as the percent involvement by giving a positive score if any pathology was seen in the quadrant (Martinez et al., 2014).

T cells, neutrophils, TMEV antigens, damaged axons, activated microglia/macrophages, and plasma cells were visualized by immunohistochemistry against CD3 (Biocare Medical, Pacheco, CA) (Sato et al., 2017), Ly-6G (BD Biosciences, San Jose, CA) (Park et al., 2020), TMEV (Nitayaphan et al., 1985), nonphosphorylated neurofilaments (SMI 311, BioLegend) (Sato et al., 2013), Iba1 (GeneTex, Inc., Irvine, CA) (Wang et al., 2021), and CD138 (BD Biosciences) (Tsunoda et al., 2005), respectively, using a Histofine MAX-PO kit (Nichirei Biosciences Inc., Tokyo, Japan) or Vector Blue Substrate kit (Vector Laboratories, Inc., Burlingame, CA). For CD3, nonphosphorylated neurofilaments, Iba1, and CD138 staining, the spinal cord sections were pretreated with a citrate buffer pH 6 (Agilent Technologies Japan, Ltd., Tokyo, Japan) for 15 minutes at 95°C using an MI-77 temperature controllable microwave (Azumaya Medical Devices Inc., Tokyo, Japan) for antigen retrieval (Sato et al., 2017). To quantify the levels of damaged axons and TMEV antigens, each spinal cord section was divided into four quadrants: the ventral funiculus, the dorsal funiculus, and each lateral funiculus. The number of SMI 311- and TMEV antigen-positive cells in each quadrant was counted under a light microscope using 10 to 14 transverse spinal cord segments per mouse, as described previously (Martinez et al., 2014).

### Lymphoproliferative Assays

In EAE, mice were killed 8–9 days post sensitization (a few days before the expected onset of disease) or 13–14 days post sensitization (the disease peak), using isoflurane (Viatris Inc., Canonsburg, PA). The inguinal lymph nodes were harvested and mashed on metal mesh with 50-μm pores using a plunger of 5-mL syringes to make single-cell suspensions. The lymph node cells were cultured in RPMI-1640 medium (Sigma-Aldrich, Co., St. Louis, MO) supplemented with 10% fetal bovine serum (FBS, Sigma-Aldrich, Co.), 2 mM L-glutamine (Sigma-Aldrich, Co.), 50 mM β-mercaptoethanol (FUJIFILM Wako Pure Chemical Corporation), 1% antibiotics (Thermo Fisher Scientific Inc., Waltham, MA) at 2 × 10^5^ cells/well in 96-well plates (Sumitomo Bakelite Co., Ltd., Tokyo, Japan) and stimulated with 50 μg/mL of the PLP_139-151_ peptide in the presence or absence of a blocking antibody against CD4 [GK1.5, American Type Culture Collection (ATCC), Rockville, MD] or CD8 (Ly2.43, ATCC) at 37°C for 5 days (Tsunoda et al., 2005). To assess the levels of PLP_139-151_-specific lymphoproliferation, we added 3 µl/well of the Cell Counting Kit-8 (CCK-8) solution (Dojindo Laboratories, Kumamoto, Japan) in the culture system for the last 24 hours (Miyamoto et al., 2002). The absorbance was measured at 450 nm using the Synergy H1 Hybrid Multi-Mode Microplate Reader (BioTek Instruments, Inc., Winooski, VT). All cultures were performed in triplicate, and the data were expressed as stimulation indexes: (mean absorbance in PLP_139-151_ stimulation)/(mean absorbance without stimulation).

In TMEV-IDD, 5–6 weeks p.i., we isolated splenic mononuclear cells (MNCs) using Histopaque^®^-1083 (Sigma-Aldrich, Co.), cultured them at 2 × 10^5^ cells/well in 96-well plates with 1 × 10^5^ cells/well of TMEV-infected antigen-presenting cells (TMEV-APCs) or mock-infected antigen-presenting cells (control-APCs) at 37°C for 5 days (Omura et al., 2018). TMEV-APCs were made from whole spleen cells infected *in vitro* with TMEV at a multiplicity of infection (MOI) of 1; control-APCs were made from mock-infected whole spleen cells (Kawai et al., 2015). Both TMEV-APCs and control-APCs were incubated overnight and irradiated with 20 Gy using an irradiator. The levels of lymphoproliferative responses to TMEV were assessed using the Carboxyfluorescein succinimidyl ester (CFSE) Cell Division Tracker Kit (BioLegend, Inc., San Diego, CA) (Barsheshet et al., 2017). All cultures were performed in triplicate, and the data were expressed as Δ% CFSE: (mean CFSE-positive cell % in TMEV-APC stimulation) − (mean CFSE-positive cell % in control-APC stimulation).

### Enzyme-Linked Immunosorbent Assays (ELISAs)

To quantify cytokines, we cultured lymph node cells from EAE mice or splenic MNCs from TMEV-infected mice at 8 × 10^6^ cells/well in 6-well plates (Sumitomo Bakelite Co.) and stimulated with 50 μg/mL of the PLP_139-151_ peptide or 5 μg/mL of concanavalin A (conA), respectively for 2 days. The culture supernatants were harvested and stored at –80°C until examined. We quantified IL-17A (BioLegend, Inc.), IFN-γ (BD Biosciences), IL-4 (BD Biosciences), and IL-10 (BD Biosciences) by ELISAs according to the manufacturers’ instructions (Martinez et al., 2014).

Using ELISAs, we also quantified serum anti-PLP_139-151_ and anti-TMEV antibody titers, as described previously (Sato et al., 2017). We coated 96-well flat-bottom Nunc-Immuno plates (Thermo Fisher Scientific Inc.) with 10 μg/ml of the PLP_139-151_ peptide or TMEV antigen. The serum samples were diluted by means of serial two-fold dilutions from 2^7^ to 2^28^ and added to the plates followed by a peroxidase-conjugated anti-mouse immunoglobulin (Ig) G1 (Thermo Fisher Scientific Inc.), IgG2c (Southern Biotechnology Associates, Inc., Birmingham, AL), IgA (Thermo Fisher Scientific Inc.), or IgM (Enzo Biochem, Inc., Farmingdale, NY) antibody (Omura et al., 2018). Immunoreactive complexes were detected with *o*-phenylendiamine dihydrochloride (FUJIFILM Wako Pure Chemical Corporation). The absorbance was measured at 492 nm using the Synergy H1 Hybrid Multi-Mode Microplate Reader. An absorbance higher than the mean + two standard deviations of naïve serum samples at a 2^7^-fold dilution was used as the standard for evaluating anti-PLP_139-151_ and anti-TMEV antibody titers.

### Real-time Polymerase Chain Reaction (Real-Time PCR)

Following perfusion with PBS, the brain and spinal cord were harvested, frozen with liquid nitrogen, and then homogenized with the TRIzol^®^ Reagent (Thermo Fisher Scientific Inc.) using a Polytron PT1200E homogenizer (Kinematica AG, Luzern, Switzerland) (Sato et al., 2017). RNA was isolated from the homogenate using the Qiagen RNeasy Mini Kit (Qiagen, Inc., Valencia, CA), according to the manufacturer’s instruction (Omura et al., 2014). We reverse-transcribed 1 µg of total RNA into cDNA using the SuperScript^®^ II Reverse Transcriptase (Thermo Fisher Scientific Inc.) (Omura et al., 2020). Using 10 ng of cDNA, real-time PCR was conducted with the THUNDERBIRD^®^ SYBR^®^ qPCR Mix (TOYOBO Co., LTD., Osaka, Japan) and the StepOnePlus^®^ Real-Time PCR System (Thermo Fisher Scientific Inc.). To determine the levels of viral replication and gene expression related to cellular and humoral immunities, adhesion molecules, and chemokines in the brain and spinal cord, we used the following primer pairs (**Table 1**): *Cxcl2*, *Cxcl9*, *Cxcl10*, *Cxcr3*, *Foxp3*, *Icam1*, *Igha*, *Il10*, *Itga4*, *Itgb2*, *Vcam1* (Eurofins Genomics, Tokyo, Japan); and *Gzmb*, *Ifng*, *Il17a* (Real Time Primers, LLC, Elkins Park, PA). A primer pair for *Pgk1* (Real Time Primers) was used as a housekeeping gene for normalization (Omura et al., 2019).

**Table 1:** The primer sets for real-time polymerase chain reaction.

Gene name	Forward primer	Reverse primer
*Cxcl2*	CCAACCACCAGGCTACAGG	GCGTCACACTCAAGCTCTG
*Cxcl9*	GGAGTTCGAGGAACCCTAGTG	GGGATTTGTAGTGGATCGTGC
*Cxcl10*	CCAAGTGCTGCCGTCATTTTC	GGCTCGCAGGGATGATTTCAA
*Cxcr3*	GGTTAGTGAACGTCAAGTGCT	CCCCATAATCGTAGGGAGAGGT
*Foxp3*	AGAGCCCTCACAACCAGCTA	CCAGATGTTGTGGGTGAGTG
*Gzmb*	TGGCCTTACTTTCGATCA AG	CAGCATGATGTCATTGGAGA
*Icam1*	GTGATGCTCAGGTATCCATCCA	CACAGTTCTCAAAGCACAGCG
*Ifng*	CAAAAGGATGGTGACATGAA	TTGGCAATACTCATGAATGC
*Igha*	TGCACAGTTACCCATCCTGA	GCACCAGCACTTCTTTAGGG
*Il10*	CTTACTGACTGGCATGAGGATCA	GCAGCTCTAGGAGCATGTGG
*Il17a*	CGCAAACATGAGTCCAGGGAGAGC	TCAGGGTCTTCATTGCGGTGGAG
*Itga4*	AACCGGGCACTCCTACAAC	CACCACCGAGTAGCCAAACAG
*Itgb2*	CAGGAATGCACCAAGTACAAAGT	GTCACAGCGCAAGGAGTCA
*Pgk1*	GCAGATTGTTTGGAATGGTC	TGCTCACATGGCTGACTTTA
*Vcam1*	TTGGGAGCCTCAACGGTACT	GCAATCGTTTTGTATTCAGGGGA

Cxcl2 [chemokine (C-X-C motif) ligand 2]; Cxcl9; Cxcl10; Cxcr3 [chemokine (C-X-C motif) receptor 3]; Foxp3 (forkhead box P3); Gzmb (granzyme B); Icam1 (intracellular adhesion molecule 1); Ifng [interferon (IFN)-γ]; Igha [immunoglobulin (Ig) heavy chain α]; Il10 [interleukin (IL)-10]; Il17a (IL-17a); Itga4 (integrin α4); Itgb2 (integrin β2); Pgk1 (phosphoglycerate kinase 1); and Vcam1 (vascular cell adhesion molecule 1).

### Principal Component Analysis (PCA)

To compare the overall clinical and immunopathological profiles between the control and curdlan treatment groups in TMEV-IDD, we conducted PCA using the clinical scores, neuropathology data, and serum anti-TMEV IgG1 and IgG2c titers 5 weeks p.i. using the “prcomp” program of R version 4.1.0, as described previously (Chaitanya et al., 2013; R Core Team, 2021). We also calculated the proportion of variance and factor loadings to determine the percentage of variance among the samples explained by each principal component (PC) and to rank the potential effectors contributing to the distribution of samples on each PC value, respectively.

### Statistical Analyses

Using the OriginPro 2020 (OriginLab Corporation, Northampton, MA), the Student’s *t*-test and Mann-Whitney *U* test were conducted for parametric data and nonparametric data, respectively. The χ^2^ test was conducted for categorical data using the GraphPad QuickCalcs (GraphPad Software, San Diego, CA, https://www.graphpad.com/quickcalcs/contingency1.cfm) (Sato et al., 2017).

## Results

### Curdlan Injection Converts RR-EAE Into Fatal Hyperacute EAE

**Figure 1:**
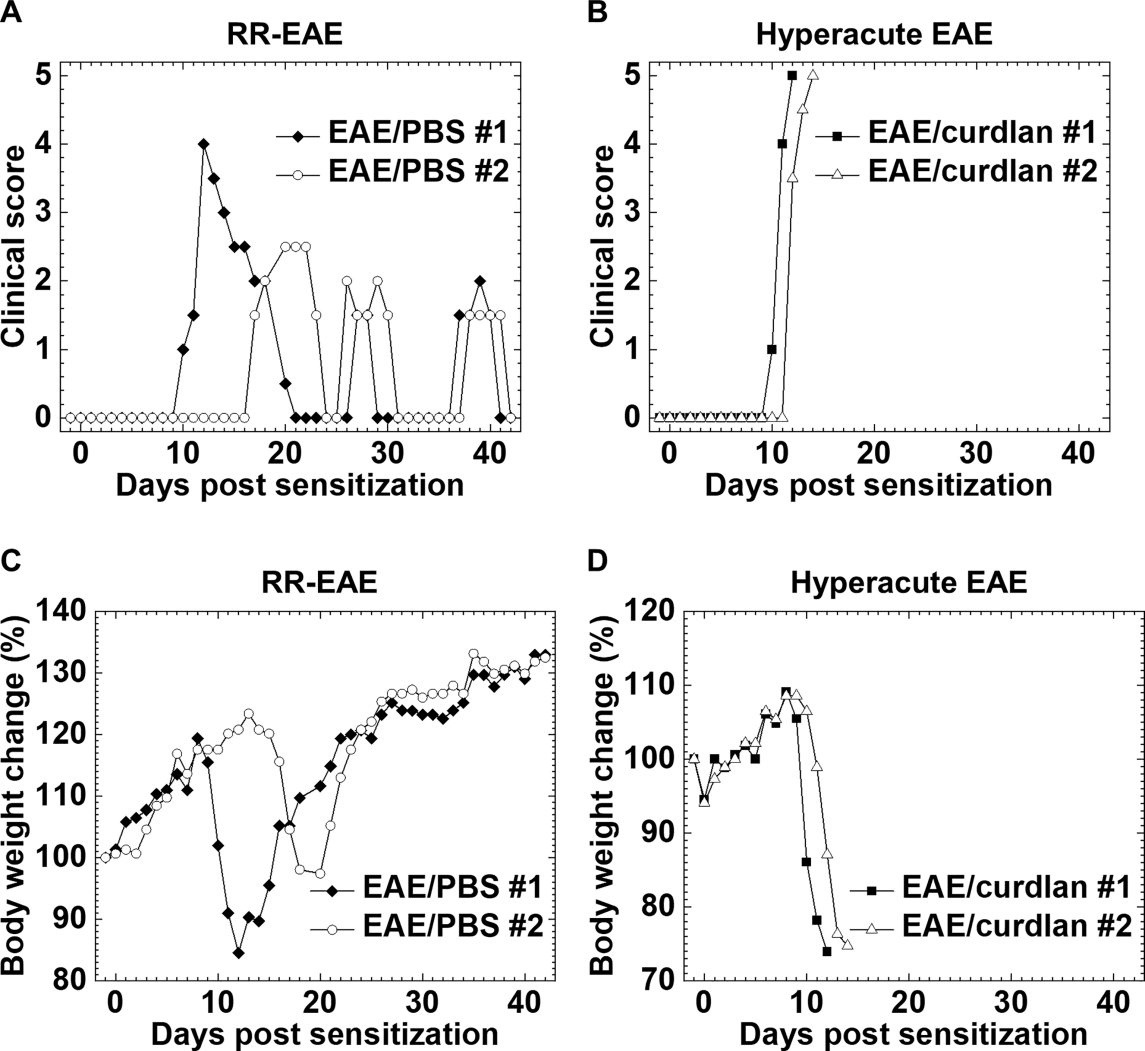
Two clinical courses of experimental autoimmune encephalomyelitis (EAE) induced with myelin proteolipid protein (PLP)_139-151_. One day prior to EAE induction, SJL/J mice were injected intraperitoneally (i.p.) with phosphate-buffered saline (PBS) (EAE/PBS, **A, C**) or curdlan (EAE/curdlan, **B, D**). Mice were observed for clinical signs **(A, B)** and weight changes **(C, D)** for 6 weeks. In the EAE/PBS group, mice developed relapsing-remitting (RR)-EAE; the mice had hind limb paralysis around 2 weeks post sensitization, recovered within 10 days (remission), and had relapses once or twice during the 6-week observation period. In the EAE/curdlan group, mice develop hyperacute EAE; the mice had progressive motor paralysis without remission and died within 1 week following the disease onset. Results are the two representative mice of 12 mice per group from three independent experiments.

We determined whether curdlan injection could affect the clinical course of EAE, an autoimmune model of MS. One day prior to subcutaneous sensitization with the PLP_139-151_/CFA, SJL/J mice were treated i.p. with curdlan or PBS. We monitored the clinical signs for 6 weeks and found that the incidence of EAE was similar between the control and curdlan treatment groups (**Table 2**). As described previously (Tsunoda et al., 1998), most PLP_139-151_-sensitized control mice developed RR-EAE; the control EAE mice developed hind limb paralysis around 2 weeks after EAE induction (mean onset day, 11.8 ± 0.7), recovered within 10 days (remission), and had relapses once or twice during the 6-week observation period (**Figure 1A**). Although the curdlan-treated EAE mice had the disease onset with hind limb paralysis (mean onset day, 12.8 ± 1.1) similar to the control EAE mice, most mice developed progressive motor paralysis without remission and became moribund or died within 20 days of EAE induction (**Table 2** and **Figure 1B**). Thus, the disease was defined as hyperacute EAE with a progressive disease course. A few mice in each group were asymptomatic or developed secondary progressive EAE (SP-EAE, **Table 2**). We also monitored the body weight changes and found that mice with RR-EAE exhibited substantial weight loss only during the first attack of EAE and gained weight thereafter regardless of relapses (**Figure 1C**). In contrast, mice with hyperacute EAE had severe progressive weight loss following the disease onset (**Figure 1D**).

**Table 2:** Incidence, mortality, and disease forms of the control mice and curdlan-treated mice in experimental autoimmune encephalomyelitis (EAE) *^a^*.

Group *^b^*	Incidence *^c^*	Mortality *^d^*	Disease forms *^e^*		
			Relapsing-remitting	Hyperacute	Secondary progressive
Control	10/12	2/10	8/10	1/10	1/10
Curdlan	11/12	9/11*	2/11	8/11	1/11

a For EAE induction, SJL/J mice were sensitized subcutaneously with the myelin proteolipid protein (PLP)_139-151_ peptide emulsified in complete Freund’s adjuvant (CFA).

b One day prior to PLP_139-151_ sensitization, mice from the control and curdlan treatment groups were injected intraperitoneally with phosphate-buffered saline (PBS) and curdlan, respectively.

c Number of symptomatic mice/total number of mice sensitized with the PLP_139-151_/CFA emulsion.

d Number of dead mice/total number of symptomatic mice. *P < 0.05, χ^2^ test, compared with controls.

e Number of mice with each disease form/total number of symptomatic mice.

### Curdlan Injection Induces Hyperacute EAE With Severe Demyelination and Massive T-Cell and Neutrophil Infiltration

**Figure 2:**
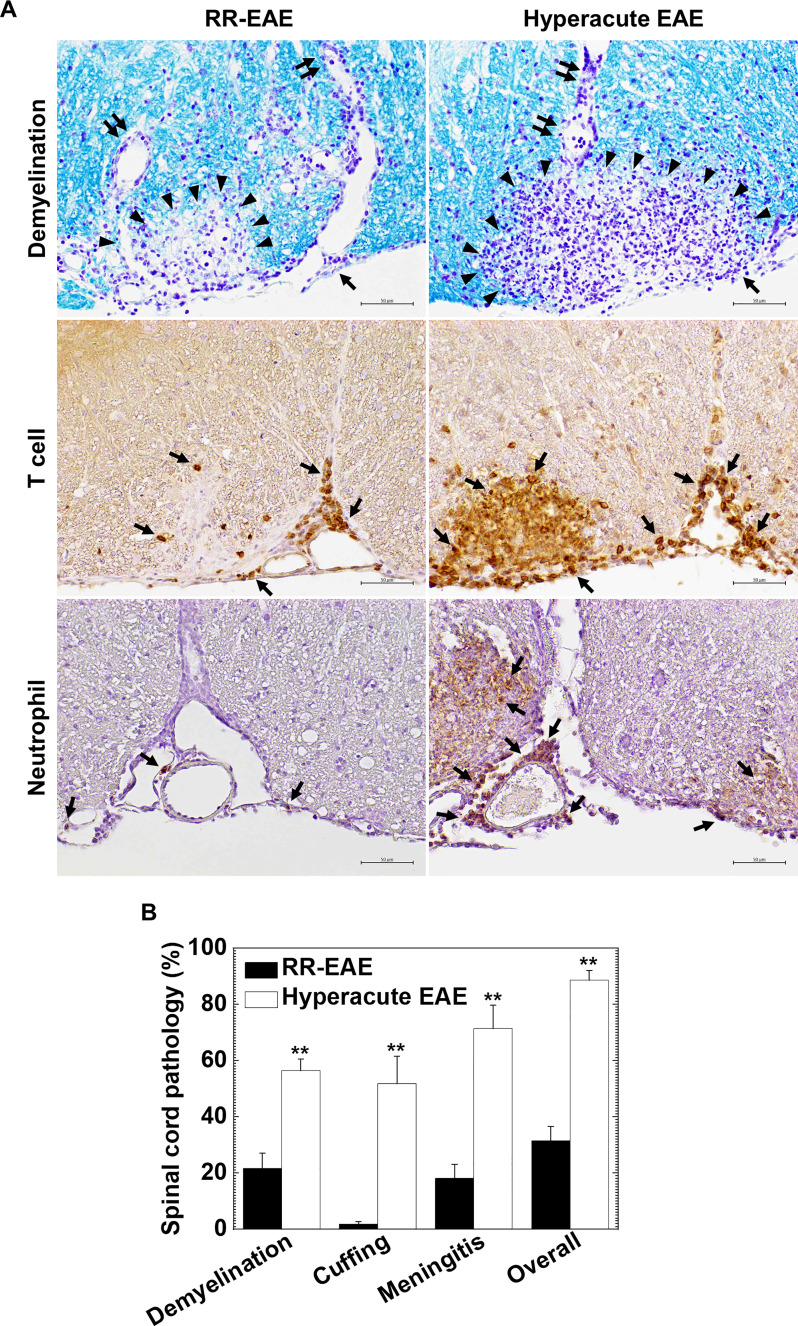
Curdlan-induced hyperacute EAE mice developed more severe demyelinating lesions with massive infiltration of T cells and neutrophils in the spinal cord. **(A)** We visualized myelin by Luxol fast blue stain (top panels) and infiltration of T cells and neutrophils by immunohistochemistry against CD3 (middle panels) and Ly-6G (bottom panels), respectively, in RR-EAE (left) and hyperacute EAE (right). In RR-EAE, we observed mild to moderate demyelination around perivascular spaces or subpial areas with infiltration of mononuclear cells (MNCs), mainly composed of CD3^+^ T cells; only a few neutrophils were observed in the meninges. In hyperacute EAE, we found severe demyelination with massive parenchymal infiltration of MNCs and polymorphonuclear cells (PMNs), which were composed of CD3^+^ T cells and Ly-6G neutrophils, respectively. In Luxol fast blue staining, arrowheads, paired arrows, and arrows indicate demyelination, perivascular cuffing (inflammation), and meningitis, respectively. In immunostaining, arrows indicate CD3^+^ T cells (middle panels) and Ly-6G^+^ neutrophils (bottom panels). Tissue sections are representative of five to eight mice per group. Scale bar = 50 μm. **(B)** Neuropathology scores of the spinal cords in RR-EAE (black bar) and hyperacute EAE (white bar). Hyperacute EAE mice had significantly higher pathology scores than RR-EAE in all pathology classes: demyelination, perivascular cuffing (inflammation), meningitis, and overall pathology. Values are the mean + standard error of the mean (SEM) of six to eight mice per group. **P < 0.01, Student’s *t*-test.

To clarify the cause of fatal hyperacute EAE following curdlan injection, we compared the neuropathology between the control and curdlan treatment groups. In the spinal cord, we found more severe inflammatory demyelination in hyperacute EAE than in RR-EAE. The spinal cord pathology scores of demyelination, perivascular cuffing, meningitis, and overall pathology were significantly higher in hyperacute EAE than in RR-EAE (*P* < 0.01, Student’s *t*-test, **Figure 2B**). In RR-EAE, immune cell infiltrates, mostly composed of MNCs, were observed in the meninges and perivascular areas (perivascular cuffing) (**Figure 2A** top panels). In contrast, in hyperacute EAE, severe demyelinating lesions contained both MNCs and neutrophils were observed not only around perivascular spaces but also in deep parenchymal areas of the spinal cord white matter. Using immunohistochemistry against CD3 (T cell marker) and Ly-6G (neutrophil marker), we found massive parenchymal infiltration of T cells (**Figure 2A** middle panels) and neutrophils (**Figure 2A** bottom panels) in hyperacute EAE. On the other hand, both RR-EAE and hyperacute EAE mice had a small number of CD138^+^ plasma cells in the meninges of the spinal cord (**Supplementary Figure 1A**). Using immunohistochemistry against nonphosphorylated neurofilaments, we also found no differences in the number of damaged axons between the two EAE forms (**Supplementary Figure 1B**). In the brain, the anatomic distribution and severity of lesions were similar between the control RR-EAE and hyperacute EAE; inflammation with or without mild demyelination was observed in the cerebellum and hippocampal fissure in both EAE forms (data not shown).

### Curdlan Injection Ameliorates Chronic TMEV-IDD, But Does Not Alter Acute Polioencephalomyelitis

**Figure 3:**
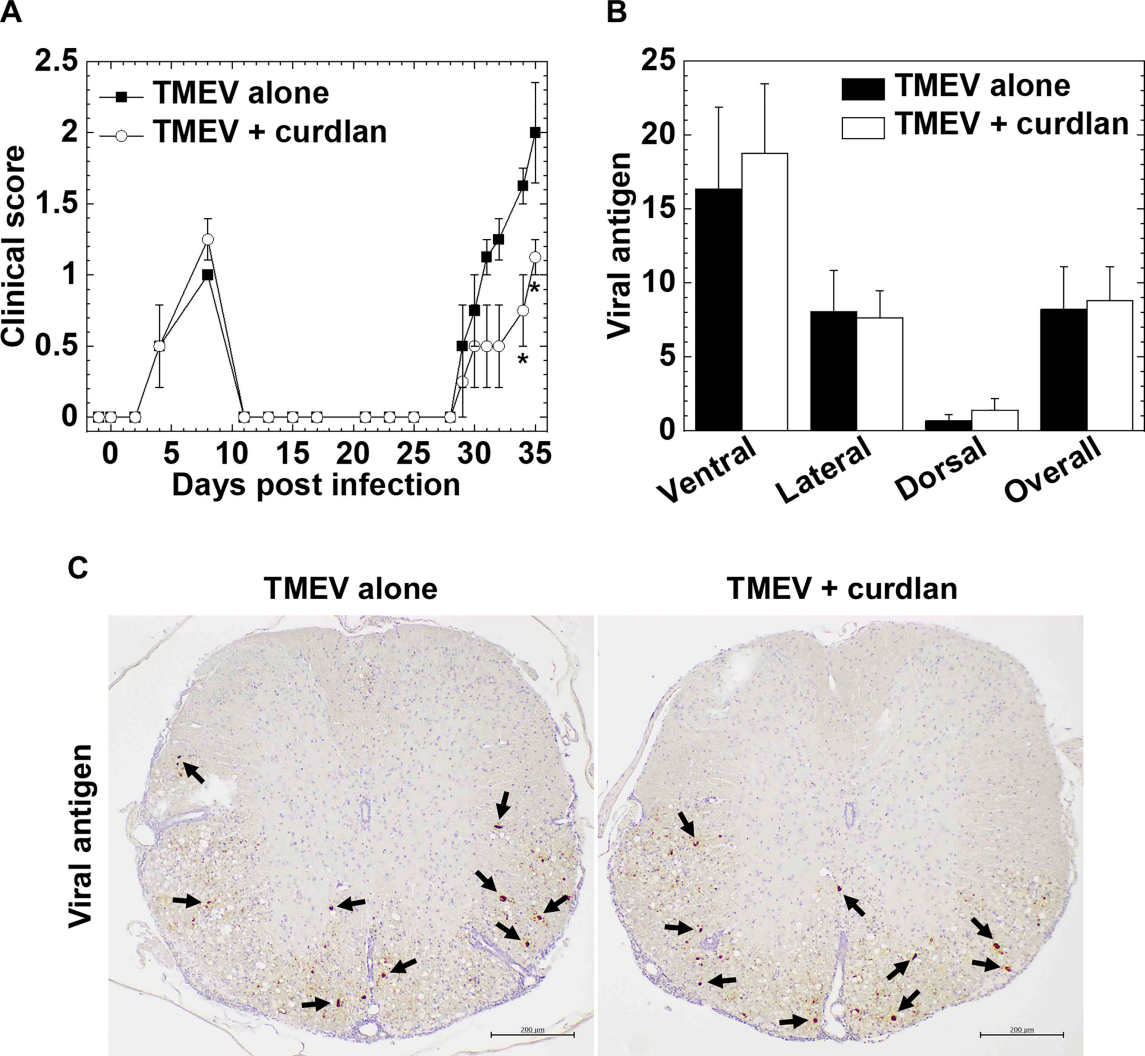
Curdlan treatment suppressed chronic Theiler’s murine encephalomyelitis virus (TMEV)-induced demyelinating disease (TMEV-IDD). **(A)** We evaluated the clinical signs by impaired righting reflex scores in the control mice (closed boxes) and curdlan-treated mice (open circles) following TMEV infection. One day prior to TMEV infection, mice were injected with curdlan (TMEV + curdlan); the control mice had no treatment (TMEV alone). The curdlan treatment group had a delayed disease onset and less severe clinical signs during the chronic phase, 1 month post infection (p.i.), but not during the acute phase, 1 week, p.i. Results are representative of three independent experiments (four mice per group per experiment). *P < 0.05, Mann-Whitney *U* test. **(B)** Numbers of viral antigen-positive cells of the spinal cord tissue sections from the control mice (black bar) and curdlan-treated mice (white bar) 5 weeks after TMEV infection. Values are the mean + SEM of four mice per group. **(C)** Immunohistochemistry against viral antigens of the spinal cord tissue sections from the control mice and curdlan-treated mice 5 weeks after TMEV infection. Arrows indicate viral antigen-positive cells (scale bar = 200 μm). Tissue sections were representative of four mice per group.

Next, we determined whether curdlan treatment could alter TMEV-IDD, a viral model of MS. We injected curdlan one day prior to TMEV infection and monitored the clinical signs and body weight changes for 5 weeks (**Figure 3A**). TMEV infection causes a biphasic disease: acute polioencephalomyelitis 1 week p.i. (acute phase) and TMEV-IDD 1 month p.i. (chronic phase). During the acute phase, TMEV infects and replicates in neurons mainly in the gray matter of the brain, leading to polioencephalomyelitis; tissue damage is induced by viral pathology, not immunopathology. During the acute phase of TMEV infection, both groups had similar impaired righting reflex scores (**Figure 3A**) and weight loss (data not shown), reflecting acute polioencephalomyelitis.

During the chronic phase, TMEV-infected mice with curdlan treatment exhibited lower clinical scores than the control TMEV-infected mice without treatment (e.g., days 34 and 35, *P* < 0.05, Mann-Whitney *U* test, **Figure 3A**). The curdlan treatment group had also delayed onset, compared with the control group [mean onset days ± standard error of the mean (SEM) of the combined data of three individual experiments: TMEV alone (n = 12), 29.5 ± 0.7; TMEV + curdlan (n = 12), 32.8 ± 0.9; *P* < 0.01, Student’s *t*-test]. In both groups, TMEV-IDD did not result in body weight loss during the chronic phase (data not shown), as described previously (Martinez et al., 2014).

### Curdlan Injection Suppresses Axonal Degeneration, But Not Inflammatory Demyelination, in TMEV-IDD

To investigate how curdlan treatment ameliorated TMEV-IDD, we compared neuropathology between the control and curdlan treatment groups. Since β-glucan treatment has been shown to play beneficial effects on several viral infections, reducing the number of viral antigen-positive cells (Jung et al., 2004; Quintin, 2019), we compared the levels of TMEV-infected cells between the two groups by immunohistochemistry with anti-TMEV antibody and quantified the viral antigen-positive cells (**Figures 3B, C**). In both groups, TMEV antigen-positive cells were mainly localized in the white matter of the ventral and lateral funiculi of the spinal cord; the numbers of TMEV antigen-positive cells were comparable.

**Figure 4:**
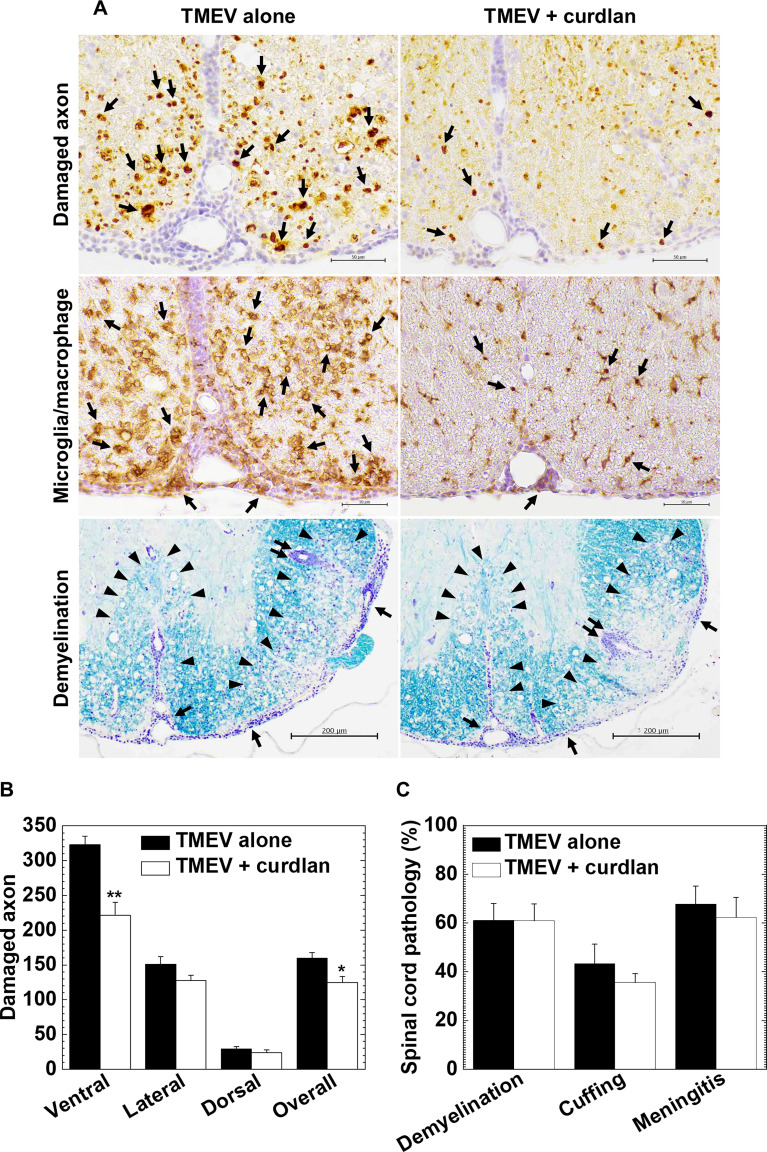
Curdlan treatment suppressed axonal damage, but not inflammatory demyelination, in TMEV-IDD. **(A)** In the curdlan treatment group, we observed fewer damaged axons (top panels) and activated macrophages/microglia (middle panels), compared with the control group. On the other hand, the levels of inflammation and demyelination were similar between the two groups (bottom panels). Immunohistochemistry against non-phosphorylated neurofilaments (top panels) and Iba1 (middle panels), and myelin stain by Luxol fast blue (bottom panels), using spinal cord sections from the control mice and curdlan-treated mice 5 weeks p.i. In immunohistochemistry (scale bar = 50 μm), arrows indicate damaged axons (top panels) and microglia/macrophage activation (middle panels). In Luxol fast blue stain (scale bar = 200 μm), arrowheads, paired arrows, and arrows indicate demyelination, perivascular cuffing (inflammation), and meningitis, respectively. Tissue sections are representative from three independent experiments (four mice per group per experiment). **(B, C)** We quantified the number of damaged axons per spinal cord quadrant **(B)** and neuropathology scores **(C)** of the spinal cord in the TMEV alone (black bar) and TMEV + curdlan groups (white bar) 5 weeks p.i. The TMEV + curdlan group had less damaged axons in the spinal cord, particularly in the ventral funiculus, compared with the TMEV alone group, although the pathology scores of demyelination, perivascular cuffing (inflammation), and meningitis were similar between the two groups. Values are the mean + SEM of four mice per group. *P < 0.05 and **P < 0.01, Student’s *t*-test.

In TMEV-IDD, axonal degeneration and inflammatory demyelination in the white matter of the spinal cord develop 1 month p.i (Tsunoda et al., 2003); axonal degeneration has been shown to contribute to clinical signs more than demyelination (Rivera-Quiñones et al., 1998; Martinez et al., 2015). Using immunohistochemistry against nonphosphorylated neurofilaments, we visualized damaged axons in the white matter of the spinal cord and compared the levels of axonal degeneration between the control and curdlan-treated mice (**Figures 4A** top panels and **4B**). TMEV-infected mice from both groups developed axonal degeneration in the white matter of the spinal cord, particularly in the ventral and lateral funiculi of the spinal cord, as described previously (Tsunoda et al., 2003). Consistent with the clinical scores, curdlan-treated mice had significantly less severe axonal degeneration in the spinal cord, particularly in the ventral funiculus, compared with the control mice (**Figure 4B**). We also visualized microglia/macrophage activation in the spinal cord by immunohistochemistry against Iba1 (microglia/macrophage marker) and found lower levels of microglia/macrophage activation in the spinal cord, particularly in the ventral funiculus (**Figure 4A** middle panels), in the curdlan treatment group than in the control group. To further determine the associations between damaged axons and activated microglia/macrophages, we conducted double-immunostaining with antibodies against nonphosphorylated neurofilaments and Iba1. We observed damaged axons adjacent to activated microglia/macrophages; TMEV-infected mice from the curdlan treatment group had fewer damaged axons and activated macrophages than the control group (**Supplementary Figure 2** top panels).

Using Luxol fast blue stain, we compared inflammation and demyelination between the control and curdlan treatment groups (**Figures 4A** bottom panels and **4C**). In both groups, we found similar levels of demyelination, perivascular cuffing (inflammation), and meningitis in the spinal cord; CNS immune infiltrates were composed of MNCs, not neutrophils, in both groups. Using immunohistochemistry against CD3, we found similar levels of T-cell infiltration in the spinal cord between the two groups (**Supplementary Figure 2** bottom panels), which was consistent with the inflammation scores in **Figure 4C**.

### Curdlan Injection Enhances Pro-inflammatory Cytokine Productions, But Not Antigen-Specific Lymphoproliferation, in EAE

**Figure 5:**
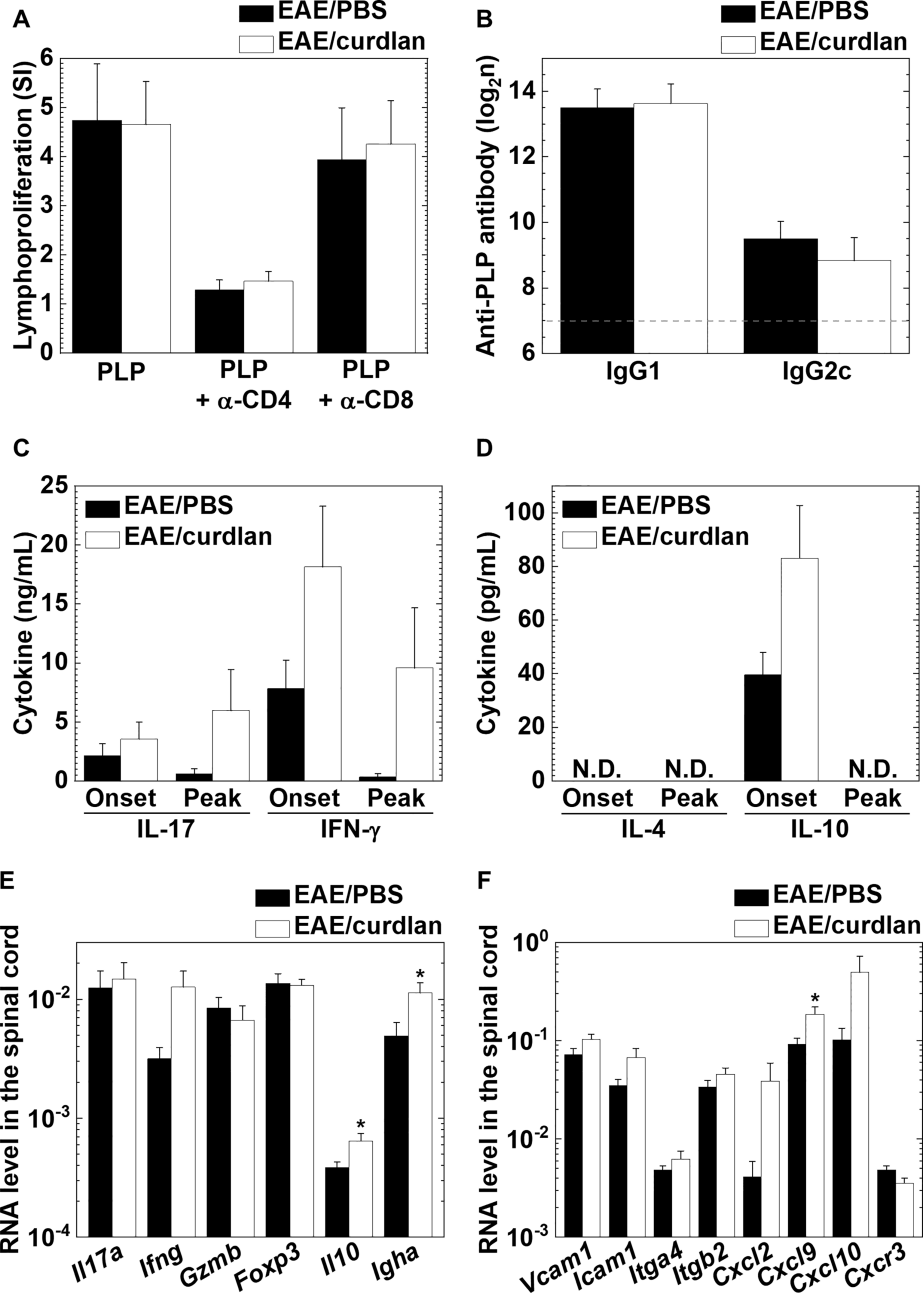
Curdlan injection enhanced the production of interleukin (IL)-17 and interferon (IFN)-γ, but not antigen-specific lymphoproliferation or antibody responses in EAE. **(A)** PLP_139-151_-specific lymphoproliferative responses of lymph node cells from the control (EAE/PBS, black bar) and curdlan-treated (EAE/curdlan, white bar) EAE mice a few days before the disease onset, 8–9 days post sensitization. Inguinal lymph node cells were stimulated with the PLP_139-151_ peptide in the presence or absence of a blocking antibody against CD4 or CD8. Levels of lymphoproliferation were expressed as stimulation indexes (SI): (mean absorbance in PLP_139-151_ stimulation)/(mean absorbance without stimulation). **(B)** Enzyme-linked immunosorbent assays (ELISAs) of serum anti-PLP_139-151_ immunoglobulin (Ig) G1 and IgG2c antibodies in sera from the control EAE and curdlan-treated EAE mice 13–14 days post sensitization. The dotted line indicates the detection limit. **(C, D)** ELISAs of IL-17, IFN-γ **(C)**, IL-4, and IL-10 **(D)** production from lymph node cells of the control EAE and curdlan-treated EAE mice harvested a few days before onset (Onset) or at the disease peak (Peak). Cells isolated from the inguinal lymph nodes were cultured with the PLP_139-151_ peptide. The four cytokines in the culture supernatants were quantified using ELISA kits. N.D., not detectable. **(E, F)** Real-time polymerase chain reaction (PCR) analyses of T cell-related **(E)** and immune cell migration/infiltration-related **(F)** genes in the spinal cord from the control and curdlan-treated EAE mice at the disease peak. *Il17a* (IL-17), *Ifng* (IFN-γ), *Gzmb* (granzyme B), *Foxp3* (forkhead box P3, regulatory T cell marker), *Il10* (IL-10), *Igha* (Ig heavy chain α, IgA marker), *Vcam1* (vascular cell adhesion molecule 1), *Icam1* (intracellular adhesion molecule 1), *Itga4* (integrin α4), *Itgb2* (integrin β2), *Cxcl2* [chemokine (C-X-C motif) ligand 2, CXCL2], *Cxcl9* (CXCL9), *Cxcl0* (CXCL10, also known as IFN-γ-inducible protein 10), and *Cxcr3* [chemokine (C-X-C motif) receptor 3, CXCR3]. *Pgk1* expression was used as a housekeeping gene for normalization. **(A–F)** Values are the mean + SEM from six to eight mice per group. *P < 0.05, Student’s *t*-test.

We determined the effects of curdlan injection on antigen-specific cellular and humoral immune responses as well as cytokine productions. In EAE, we found no differences in the levels of PLP_139-151_-specific lymphoproliferation between the control and curdlan treatment groups (**Figure 5A**). The lymphoproliferative responses were suppressed in the presence of a blocking antibody against CD4, but not CD8, in the two groups; we confirmed the previous findings (Tsunoda and Fujinami, 1996) that PLP_139-151_ sensitization primed only CD4^+^ T cells. Similarly, curdlan injection did not alter serum anti-PLP_139-151_ IgG1 or IgG2c antibody titers (**Figure 5B**). Furthermore, we examined whether curdlan injection could affect antigen-specific cytokine profiles in the periphery using the regional lymph node cells a few days before the disease onset (day 8 or 9) and at the disease peak (day 13 or 14). We quantified the amounts of pro-inflammatory IL-17/IFN-γ and anti-inflammatory IL-4/IL-10 cytokines by cytokine ELISAs (**Figures 5C, D**). Before the onset of EAE, the levels of IL-17, IFN-γ, and IL-10 production were higher in the curdlan treatment group than in the control group. At the disease peak, IL-17 and IFN-γ levels, but not IL-10, remained high in the curdlan group, although IL-17 and IFN-γ levels were decreased substantially in the control group. IL-4 was undetectable in both groups. We also determined the effects of curdlan on major immune-related gene expressions, including cytokines/chemokines, transcription factors, and adhesion molecules, in the spinal cord at the disease peak by real-time PCR. Compared with the control EAE mice, the curdlan-treated EAE mice had significantly higher levels of IL-10, IgA, and CXCL9 gene expressions in the spinal cord (*P* < 0.05, Student’s *t*-test); the levels of IFN-γ, VCAM-1, ICAM-1, and CXCR3 gene expressions tended to be higher in the spinal cord of curdlan-treated mice (*P* < 0.1, Student’s *t*-test, **Figures 5E, F**).

### Curdlan Injection Does Not Alter TMEV-Specific Lymphoproliferation or Expressions of Cytokine/Adhesion Molecules in TMEV Infection

**Figure 6:**
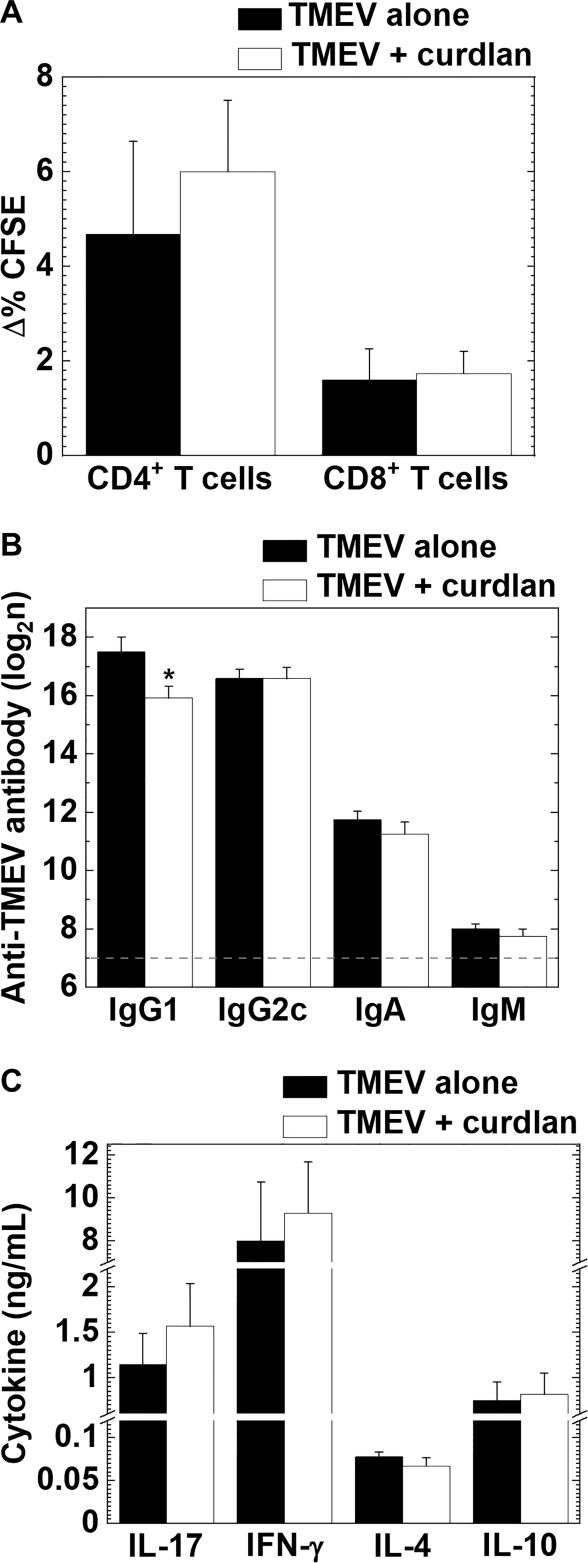
TMEV-specific T cell proliferation and antibody responses, and cytokine productions in TMEV-IDD with or without curdlan treatment. **(A)** We found similar levels of TMEV-specific CD4^+^ T cell and CD8^+^ T cells responses between the control (TMEV alone, black bar) and curdlan-treated (TMEV/curdlan, white bar) mice 5–6 weeks p.i. Values of TMEV-specific lymphoproliferative responses were expressed as Δ% CFSE: (mean CFSE-positive cell % in TMEV-APC stimulation) − (mean CFSE-positive cell % in control-APC stimulation). **(B)** Although the curdlan treated group had lower serum anti-TMEV IgG1 titers compared with the control group, anti-TMEV IgG2c, IgA, and IgM titers were similar between the two groups 5–6 weeks p.i. The dotted line indicates the detection limit. *P < 0.05, Student’s *t*-test. **(C)** IL-17, IFN-γ, IL-4, and IL-10 production from splenic MNCs of the control and curdlan-treated mice 5–6 weeks p.i. Antibody titers and cytokine concentrations were determined by ELISAs. **(A–C)** Values are the mean + SEM from eight to twelve mice per group.

In TMEV-IDD, we compared the levels of TMEV-specific lymphoproliferative responses between the TMEV alone and TMEV + curdlan groups and found that both TMEV-specific CD4^+^ and CD8^+^ T cell responses were comparable between the two groups (**Figure 6A**). We also compared serum anti-TMEV antibody titers and cytokine profiles by ELISAs between the two groups. The curdlan treatment group had significantly reduced anti-TMEV IgG1 (induced by IL-4) titers, compared with the TMEV alone group, although there were no differences in anti-TMEV IgG2c (induced by IFN-γ), IgA, or IgM titers (**Figure 6B**). On the other hand, curdlan injection enhanced the amounts of IL-17 and IFN-γ, but not the amounts of IL-4 or IL-10, although there were no statistical differences between the two groups (**Figure 6C**).

**Figure 7:**
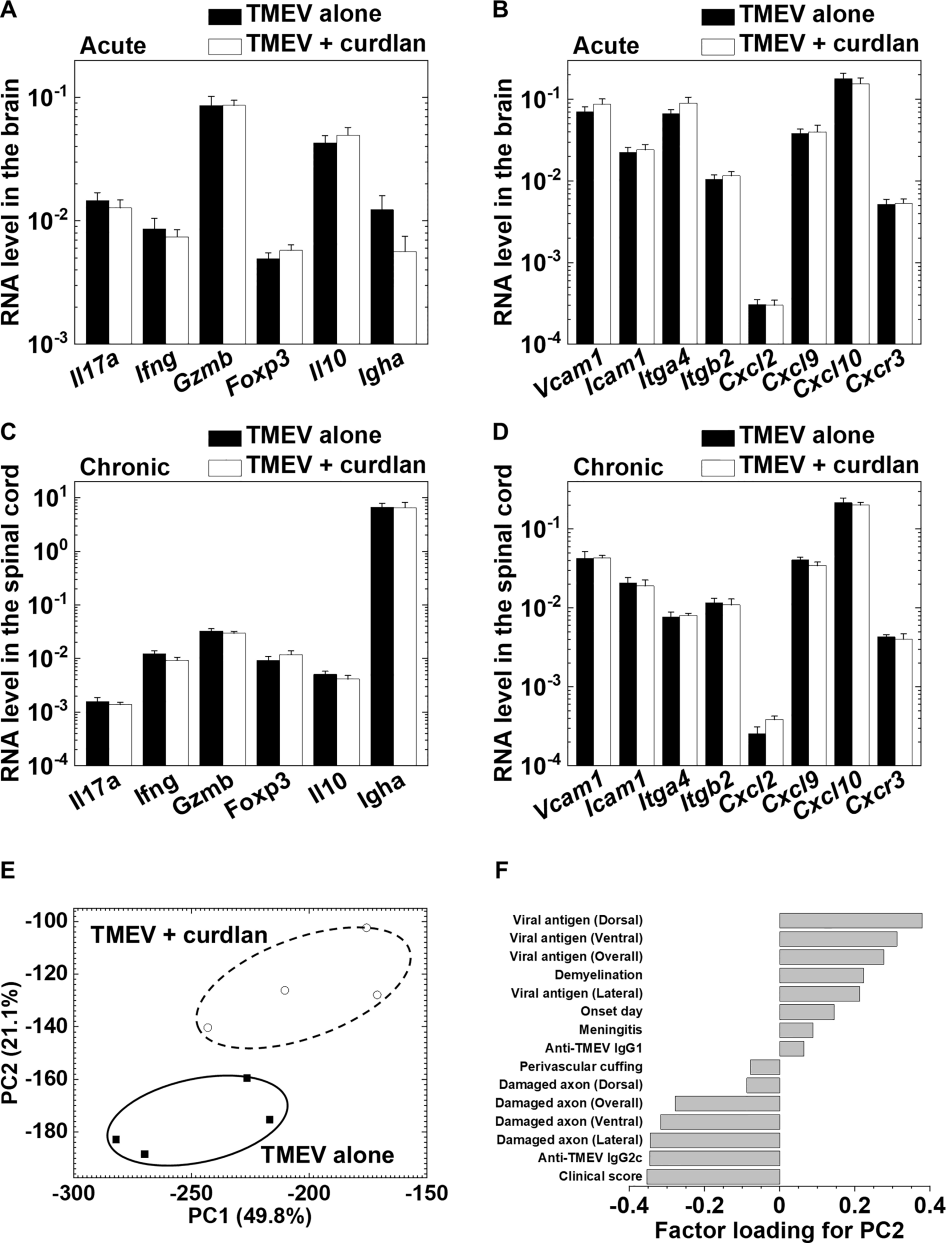
Curdlan injection did not alter immune-related gene expressions in the central nervous system (CNS) during the acute and chronic phases of TMEV infection between the control and curdlan treatment groups. **(A–D)** Real-time PCR analyses of T cell-related and immune cell migration/infiltration-related genes in the brain 8 days p.i. **(A, B)** and in the spinal cord 5 weeks p.i. **(C, D)**. **(A, C)** mRNA levels of *Il17a*, *Ifng*, *Gzmb*, *Foxp3*, *Il10*, and *Igha*. **(B, D)** mRNA levels of *Vcam1*, *Icam1*, *Itga4*, *Itgb2*, *Cxcl2*, *Cxcl9*, *Cxcl0*, and *Cxcr3*. *Pgk1* expression was used as a housekeeping gene for normalization. Values are the mean + SEM of four mice per group. **(E)** Principal component analysis (PCA) separated TMEV-infected control and curdlan-treated mice as two distinct populations by the principal component (PC) 2 values. We conducted PCA, using 15 immunopathology data: neuropathology (demyelination, perivascular cuffing, meningitis, viral-antigen positive cells, damaged axons), antibody titers, clinical scores, and the disease onset day from the two groups 5 weeks p.i. Each group was composed of four mice. **(F)** Factor loading for PC2 showed that clinical scores and damaged axon numbers contributed to the PC2 distribution.

We also determined the effects of curdlan on immune cells/molecules in the CNS during the acute and chronic phases of TMEV infection by real-time PCR analyses of gene expressions of T-cell related molecules, cytokines/chemokines, and adhesion molecules. We found no differences in the levels of cellular/humoral immunity-related gene expressions or chemokine/adhesion molecule gene expressions during the acute phase (**Figures 7A, B**), or during the chronic phase of TMEV infection between the control and curdlan treatment groups (**Figures 7C, D**).

Although we found no differences in immune-related gene expressions between the control and curdlan treatment groups, we found statistical differences in clinical signs, damaged axons, and immunoglobulin titers. PCA has been used to characterize the overall pathophysiology of disease conditions from multivariate data that can be composed of a different set of metrics (Sato et al., 2014; Omura et al., 2019). By calculating factor loading for PC values, one can rank the multivariate data based on the extent of which components contribute to each PC value. Thus, we used the clinical, neuropathological, and immunological data to characterize the overall pathophysiology associated with curdlan treatment. PCA clearly separated the two groups into two distinct populations by PC2 values, which seemed to reflect the disease severity; the proportion of variance of PC1 and PC2 were 49.8% and 21.1%, respectively (**Figure 7E**). Factor loading for PC2 showed that the clinical and axonal damage scores contributed to PC2 values, although viral persistence and inflammatory demyelination were not associated with PC2 values (**Figure 7F**). This was consistent with the previous findings that axonal damage, rather than the extent of viral persistence or inflammatory demyelination, contributed to clinical disability in TMEV-IDD (Yamada et al., 1990; Rivera-Quiñones et al., 1998).

## Discussion

In this study, we demonstrated that microbial component curdlan injection in the autoimmune and viral models for MS resulted in disease exacerbation and amelioration, respectively. In the PLP_139-151_-induced RR-EAE model, a pure CD4^+^ T cell-mediated disease, curdlan injection prior to PLP sensitization induced hyperacute EAE and the mice died around 2 weeks after EAE induction. In TMEV-IDD, curdlan injection prior to TMEV infection delayed the disease onset and reduced the severity of chronic disease, around 1 month p.i. On the other hand, we previously reported that curdlan injection exacerbated MOG_92-106_-induced EAE in SJL/J mice, who died around 1 month after EAE induction, where enhancement of CD4^+^ T-cell and anti-MOG demyelinating antibody responses converted RR-EAE into chronic primary progressive (PP)-EAE (Omura et al., 2019). Thus, the effects of curdlan injection on MS models seemed to be different, depending on several factors such as the etiology (autoimmune versus viral), disease onset (acute versus chronic disease), and effector mechanism (myelin-specific CD4^+^ T cells, anti-myelin antibodies, or anti-viral immune responses). We also found that curdlan injection on day 21 after the onset of PLP_139-151_-induced RR-EAE (**Supplementary Figure 3**) or on day 53 after the onset of MOG_92-106_-induced EAE (Libbey et al., 2010) converted the RR disease course into a progressive course in more than 50% of EAE mice. On the other hand, using C57BL/6 mice, we determined the effects of curdlan in MOG_35-55_-induced EAE, in which EAE mice have a monophasic disease course without relapsing (Fernando et al., 2014). Although curdlan injection in the MOG_35-55_-induced EAE model developed hyperacute EAE with similar neuropathology to what was observed in the PLP_139-151_-induced EAE model, the incidence of hyperacute EAE was lower in MOG_35-55_-induced EAE mice than in PLP_139-151_-EAE mice (**Supplementary Table 1**). On the other hand, most curdlan-injected C57BL/6 mice developed monophasic EAE without differences in the clinical signs or the mortality, compared with PBS-injected control EAE mice. Thus, the effects of curdlan injection differed between the two classic EAE models: the MOG_35-55_-induced EAE and PLP_139-151_-induced EAE models. Although it is unclear what factors contribute to the different outcomes between the two EAE models, both models have several differences that may play a role. For example, to induce EAE, injection of pertussis toxin, which has been proposed to act as an adjuvant and break the blood-brain barrier, is required for C57BL/6 mice, but not SJL/J mice. C57BL/6 mice develop monophasic EAE without relapses; SJL/J mice develop RR-EAE. SJL/J mice have several immunological and genetical deficits, including the absence of one major histocompatibility complex class II molecule, several TCR repertoire, and natural killer (NK) cells (Kaminsky et al., 1985; Friedmann et al., 1987; Bahk et al., 1997); both mouse strains also have different compositions of the gut microbiota that has been shown to modulate immune responses (Gandy et al., 2019). These findings give insight into the inconsistent and anecdotal reports on the involvement of bacterial and fungal infections in MS published previously; immunomodulation by microbial components likely depends on the immunological and etiological background of each MS patient.

In this study, to gain mechanistic insight, we tested whether curdlan could directly stimulate encephalitogenic effector cells, conferring the ability to induce hyperacute EAE. We used two passive EAE models, where MOG_35-55_- or PLP_139-151_-specific T cells were activated *in vitro* in the presence or absence of curdlan and then transferred into naive C57BL/6 or SJL/J mice, respectively. In the two passive EAE models, however, *in vitro* direct stimulation of encephalitogenic effector cells with curdlan neither increased the incidence of EAE nor induced hyperacute EAE (**Supplementary Table 2**). Thus, the direct stimulation of effector encephalitogenic cells with curdlan alone was not sufficient for hyperacute EAE induction.

In curdlan-induced hyperacute EAE, we found massive infiltration of both T cells and neutrophils in the spinal cord, not in the brain. The heavy neutrophil infiltration in the CNS was similar to the classical hyperacute EAE model in rats (Levine and Wenk, 1965), although the hemorrhage observed in the classical hyperacute EAE was rare in the curdlan-induced hyperacute EAE. Neutrophil infiltration has also been observed in other PP-EAE models, including MOG_92-106_-induced PP-EAE, where IL-17-producing Th17 cells play a role in the neutrophil recruitment in the CNS (Omura et al., 2019). In this study, we found that the levels of IL-17 and IFN-γ production remained high at the peak of hyperacute EAE, but not RR-EAE, suggesting the pathogenic roles of pro-inflammatory Th17 cells and Th1 cytokines in disease progression. This was consistent with the findings that curdlan has been known as a Th17 inducer; curdlan has also been shown to enhance Th1 responses (LeibundGut-Landmann et al., 2007; Kim et al., 2016). Although the precise pathomechanism of curdlan-mediated Th1 responses is still controversial, curdlan has been shown to be recognized by toll-like receptor 4, resulting in the increased production of IL-12, which contributes to Th1 differentiation (Kim et al., 2016). Alternatively, Th17 cells have been reported to trigger the recruitment of Th1 cells by inducing chemokines, such as CXCL9, CXCL10, and CXCL11. In this study, we found that the expression levels of *Cxcl9*, *Cxcl10*, and *Ifng* in the spinal cord were higher in the curdlan-treated EAE mice than in the control EAE mice. Furthermore, curdlan treatment enhanced the gene expressions of adhesion molecules including *Vcam1* and *Icam1*; PCA showed that the increased expressions of *Cxcl9* and *Cxcl10* as well as *Cxcl2* (neutrophil chemokine) in the spinal cord could contribute to immunopathology of the curdlan treatment group (data not shown). These results were consistent with the neuropathological findings that the curdlan treatment EAE group had more severe CNS inflammation and higher levels of myelin-specific Th17 and Th1 responses than the control EAE group. On the other hand, we found no changes in anti-PLP antibody responses or axonal degeneration in hyperacute EAE mice, suggesting that these factors have no roles in disease exacerbation in the curdlan treatment group.

Curdlan has also been reported to enhance IL-10 production and favor IgA antibody responses (LeibundGut-Landmann et al., 2007; Kawashima et al., 2012; Fujimoto et al., 2019). Recently, IgA^+^ cells have been detected in the active demyelinating lesions of MS patients (Probstel et al., 2020); IgA-secreting plasma cells have been shown to play protective roles in EAE by producing IL-10 (Rojas et al., 2019). Thus, we had anticipated that curdlan treatment might be beneficial in our EAE model. However, this was not the case. Although we found significantly increased levels of IL-10 and IgA gene expression in the spinal cord of curdlan-treated EAE mice, we did not see increases in IgA-producing cells (data not shown) or CD138^+^ plasma cells (**Supplementary Figure 1**) in the spinal cord by immunohistochemistry in the curdlan treatment group.

In contrast to EAE, curdlan injection ameliorated TMEV-IDD with reduced axonal degeneration in the spinal cord, although it did not alter the levels of inflammation/demyelination or viral persistence. This was consistent with the previous findings by Rivera-Quiñones et al. (1998) that the clinical signs of TMEV-IDD were associated with axonal degeneration rather than inflammatory demyelination. Previously, we also demonstrated that, although TMEV-infected SJL/J and RORγt transgenic mice had similar levels of inflammatory demyelination, only TMEV-infected SJL/J mice had neurological signs with severe axonal damage. Here, TMEV-infected RORγt transgenic mice had no clinical signs because of significantly reduced axonal degeneration (Martinez et al., 2015).

Several factors have been suggested to contribute to axonal degeneration in TMEV-IDD, for example, activation of microglia/macrophages, CD8^+^ T cells, and neuronal damage during the acute phase of TMEV infection. In this study, we found that Iba1^+^ activated microglia/macrophages accumulated around the lesions with damaged axons in the spinal cord. Since β-glucan treatment has been shown to induce a long-lasting immunomodulatory effect on innate immune cells including microglia (Haley et al., 2019), curdlan may affect microglia/macrophages by binding to the specific receptor dectin-1 during the early phase of TMEV infection, which influences the activation status of microglia/macrophages during the chronic phase of TMEV infection, 1 month p.i. Using immunohistochemistry against dectin-1 (Dong et al., 2014), we examined the expression levels of dectin-1 in the spinal cord as well as in the spleen as the positive control. Consistent with previously published data (Dong et al., 2014), we found a small number of dectin-1-positive cells in the spleen, but not in the spinal cord (data not shown). This could be due to the low sensitivity limit used in the current study; the associations between dectin-1 expression and activated microglia and macrophages remain unknown. Although CD8^+^ T cells have also been proposed to damage axons in TMEV infection, this seemed not to be the case in the curdlan treatment group; we did not find increased levels of CD8^+^ T cell responses to TMEV or the upregulation of *Cd8a* (data not shown) and *Gzmb* in the spinal cord. Alternatively, the early neuronal damage by lytic TMEV infection during the acute phase can alter the levels of axonal degeneration later on; neuronal and axonal damage can be followed by Wallerian degeneration of axons. This is also unlikely since we found no difference in the severity of acute polioencephalomyelitis or CNS viral replication during the acute phase (data not shown) between the control and curdlan treatment groups.

In TMEV-IDD, TMEV-specific CD4^+^ and CD8^+^ T cells, as well as anti-TMEV antibodies, have been demonstrated to contribute to inflammatory demyelination (Sato et al., 2018). In this study, we did not see differences in TMEV-specific CD4^+^ and CD8^+^ T cell proliferation or anti-TMEV isotype responses between the control and curdlan treatment groups, except for the mild reduction of IgG1 titers in the curdlan treatment group. Since Th2 responses help immunoglobulin class switching to IgG1 in mice, the reduced anti-TMEV IgG1 titers in the curdlan treatment group can be due to the increased IFN-γ (Th1 cytokine)/IL-4 (Th2 cytokine) ratios (mean IFN-γ/IL-4 ± SEM: TMEV alone, 108.0 ± 39.5; TMEV + curdlan, 151.6 ± 46.2), although it did not reach statistical difference. We also found similar expression levels of Th-related cytokines, *Il17*, *Ifng*, and *Il10*, adhesion molecules, *Vcam1*, *Icam1*, *Itga4*, and *Itgb2*, and chemokines/chemokine receptor, *Cxcl2*, *Cxcl9*, *Cxcl10*, and *Cxcr3*, in the spinal cord between the control and curdlan treatment groups. These immune profiles were consistent with similar levels of inflammatory demyelination between the two groups.

In summary, we demonstrated that curdlan injection was detrimental in an autoimmune model for MS by converting it into a fatal hyperacute EAE with massive infiltration of T cells and neutrophils. On the other hand, curdlan injection ameliorated a viral model for MS with decreased CNS axonal degeneration, although it did not alter inflammation or demyelination. Since human MS has been suggested to be heterogenous with different etiology and immunopathology depending on each patient, the effects of microbial immunomodulating components, including curdlan, in human MS may differ. This could reflect diverse and often conflicting etiological reports of human MS following bacterial and fungal infections. It has been reported that, depending on the disease stage or subtype of MS, neuropathology of MS can be predominantly composed of axonal degeneration with microglia/macrophage activation; curdlan-like microbial components might be useful to treat such MS neuropathology. Lastly, our study supports the view that MS is a heterogeneous disease whose treatment needs to be tailored on a more individual basis, rather than considering only whether MS patients have an RR or progressive course. Our study also raises the issue that the clinical courses of MS may depend on the type of exposure (bacteria/virus/fungus), although it should be clarified whether other microbial components, such as *Mycobacterium smegmatis* (Kannan et al., 2020), also affect the clinical courses of MS, as a single modulator or together with other microbial components. In the future, these experimental and clinical approaches would lead to develop a basis for therapy, for example, to determine the levels of β-glucan as a biomarker in circulation might be beneficial once its role is clarified.

## Data Availability Statement

The raw data supporting the conclusions of this article will be made available by the authors, without undue reservation.

## Ethics Statement

The animal study was reviewed and approved by Institutional Animal Care and Use Committee of Kindai University Faculty of Medicine and LSUHS.

## Author Contributions

FS and IT designed the experiments and prepared the manuscript. FS, YN, AK, SK, and IA performed the experiments. NEM and SO aided in the experiments and manuscript preparation. IT supervised the experiments. All authors read and approved the final manuscript.

## Funding

This work was supported by the Institutional Development Award (IDeA) from the National Institute of General Medical Sciences of the NIH (5P30GM110703, IT), the fellowships (FS and SO) from the Malcolm Feist Cardiovascular Research Endowment, LSUHS, the Research Program on Emerging and Re-emerging Infectious Diseases from the Japan Agency for Medical Research and Development (AMED, 21fk0108084h0803, IT), the All-Kindai University Support Project against COVID-19 (IT), and the KAKENHI from the Japan Society for the Promotion of Science [Grant-in-Aid for Scientific Research (C): JP20K07433, FS; JP19K08569, SO; and JP20K07455, IT].

## Conflict of Interest

The authors declare that the research was conducted in the absence of any commercial or financial relationships that could be construed as a potential conflict of interest.

## Publisher’s Note

All claims expressed in this article are solely those of the authors and do not necessarily represent those of their affiliated organizations, or those of the publisher, the editors and the reviewers. Any product that may be evaluated in this article, or claim that may be made by its manufacturer, is not guaranteed or endorsed by the publisher.
