# Supplementary material for: Curdlan, a Microbial β-Glucan, Has Contrasting Effects on Autoimmune and Viral Models of Multiple Sclerosis
Source: Front Cell Infect Microbiol. 2022 Feb 7;12:805302. doi: 10.3389/fcimb.2022.805302 (PMC8859099; doi:10.3389/fcimb.2022.805302)
Supplement: Supplementary file 1 [file DataSheet_1.pdf]

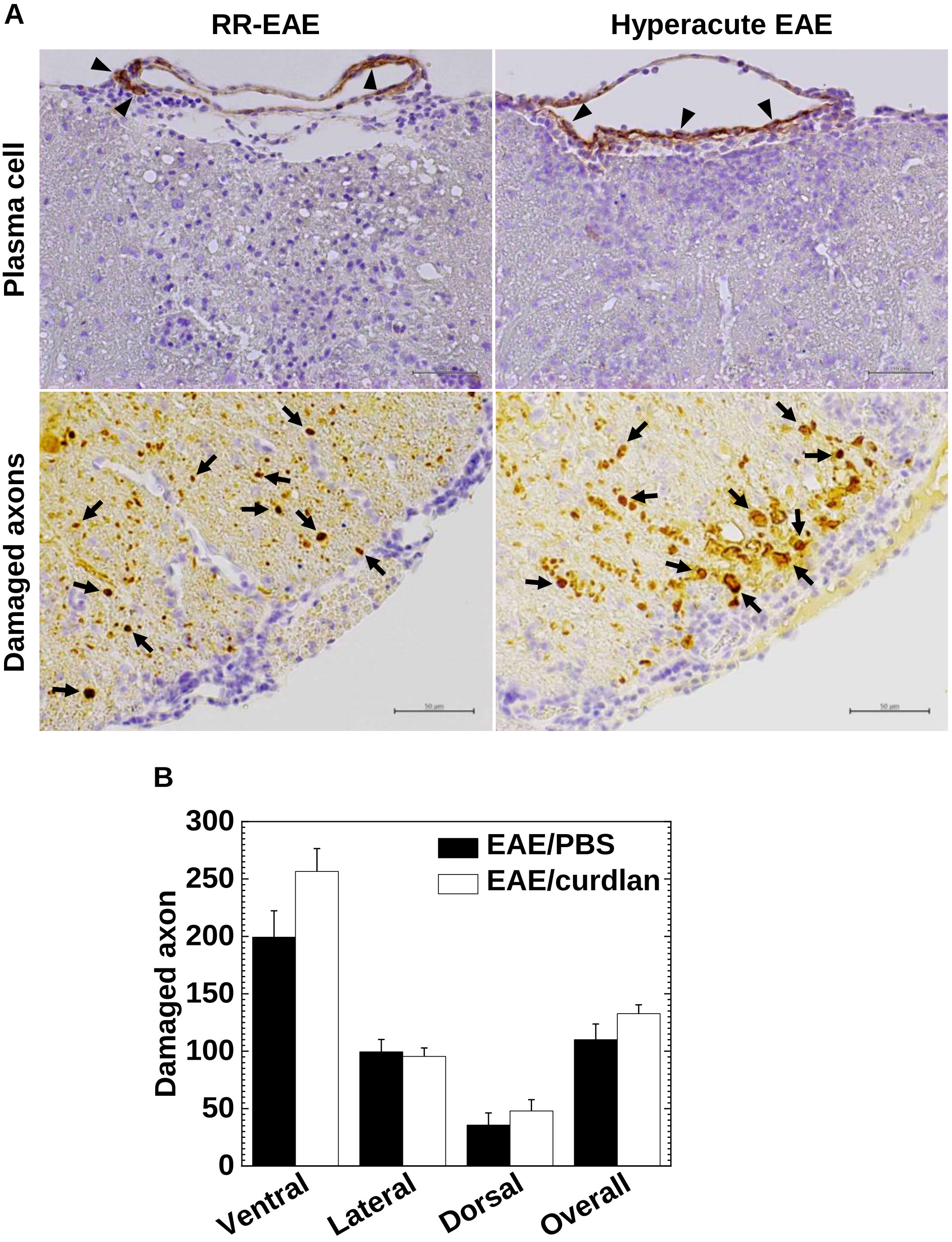

Supplementary Figure 1. Curdlan injection did not alter CD138-positive cell infiltration or axonal damage in the spinal cord of mice with myelin proteolipid protein $(PLP)_{139-151}$-induced experimental autoimmune encephalomyelitis (EAE). (**A**) Immunohistochemistry against CD138 and nonphosphorylated neurofilaments of the spinal cord tissue sections from relapsing-remitting EAE (RR-EAE) and curdlan-induced hyperacute EAE mice. Arrowheads and arrows indicate CD138-positive cells (scale bar = 200 μm) and damaged axons (scale bar = 50 μm), respectively. Tissue sections are representative of five to eight mice per group. (**B**) Number of damaged axons in each quadrant of the spinal cord sections from RR-EAE (black bar) and curdlan-induced hyperacute EAE (white bar) mice. Values are the mean + standard error of the mean (SEM) of five mice per group.

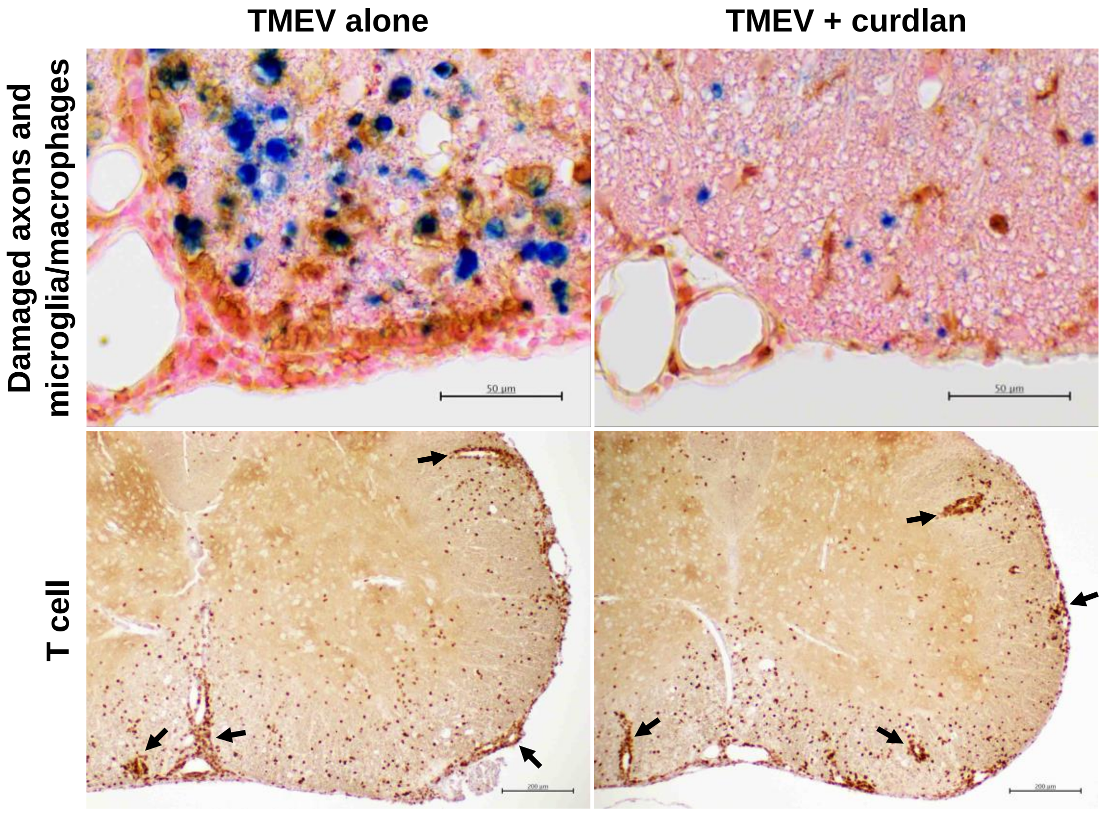

Supplementary Figure 2. Curdlan injection reduced damaged axons and activated microglia/macrophages, but not T cells, in the spinal cord 5 weeks after Theiler's murine encephalomyelitis virus (TMEV) infection. We conducted double-immunostaining to determine the associations between damaged axons (blue) and activated microglia/macrophages (brown, top panels, scale bar = 50 μm). We also visualized T cells by immunohistochemistry with anti-CD3 antibody. Arrows indicate CD3-positive T cells (bottom panels, scale bar = 200 μm). Tissue sections are representative of four mice per group.

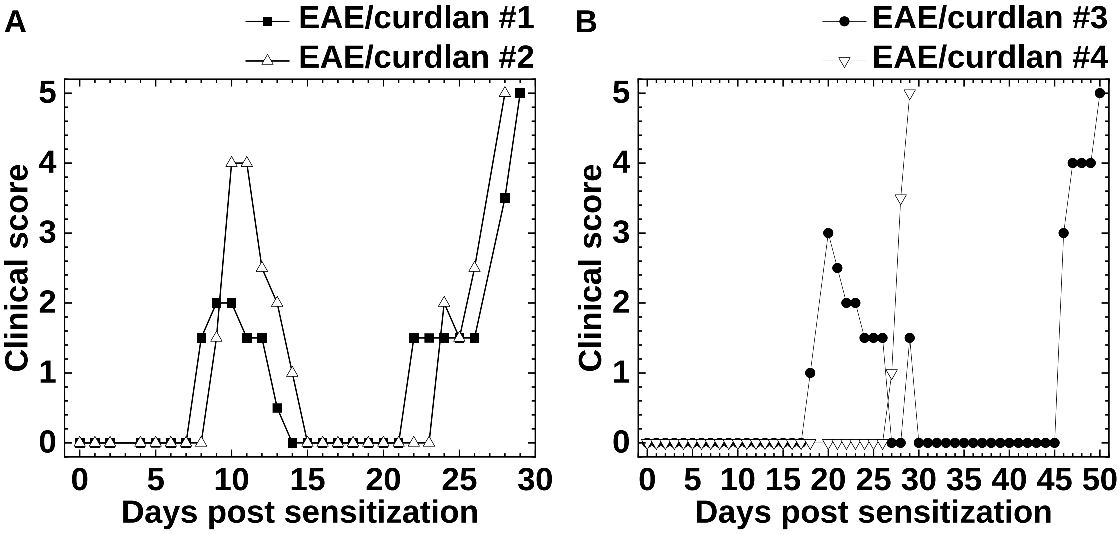

Supplementary Figure 3. Curdlan injection converted a relapsing-remitting form of EAE into a fatal progressive form. SJL/J mice were sensitized with the $PLP_{139\text{-}151}$ peptide emulsified in complete Freund's adjuvant (CFA) and injected intraperitoneally with curdlan 21 days after EAE induction. In the three independent experiments, 73% of curdlan-injected EAE mice (11 out of 15 mice) had fatal progressive disease courses. (**A, B**) Representative clinical courses of a fatal progressive EAE following curdlan injection on day 21.

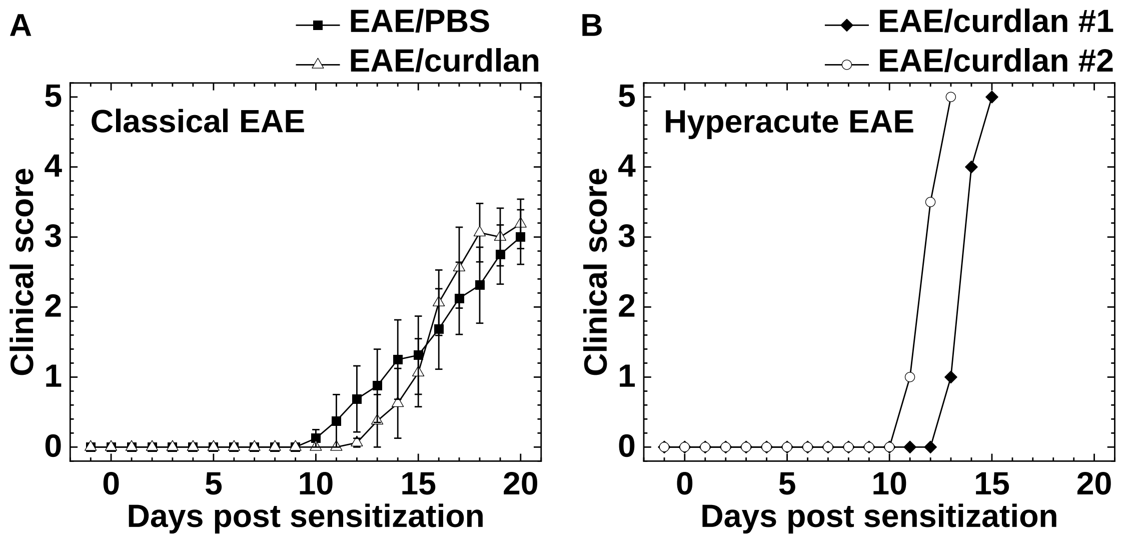

Supplementary Figure 4. Curdlan injection converted a monophasic form of EAE into a hyperacute form. C57BL/6 mice were injected intraperitoneally with phosphate-buffered saline (PBS) or curdlan (5 μg/mouse). One day after treatment, mice were sensitized subcutaneously with the myelin oligodendrocyte protein $(MOG)_{35\text{-}55}$ peptide emulsified in CFA. We monitored their clinical signs daily. (**A**) All mice from the control group developed classical EAE. On the other hand, in the curdlan treatment group, eight out of ten mice (80%) exhibited classical EAE (**A**); two out of ten mice (20%) had hyperacute EAE (**B**). Each group was composed of eight to ten mice.

Supplementary Table 1. Effects of curdlan in $MOG_{35\text{-}55}$-induced active EAE.

Group	EAE type	Incidence	Onset day	Maximum score	Mortality (%)
PBS	Classical	8 / 8	15.0 ± 1.2	3.1 ± 0.4	0 / 8 (0%)
Curdlan	Classical	8 / 10	15.1 ± 0.6	3.3 ± 0.0	0 / 8 (0%)
Curdlan	Hyperacute	2 / 10	12.0 ± 1.0	5.0 ± 0.0	2 / 2 (100%)

Supplementary Table 1. We determined whether curdlan injection could induce a hyperacute form in $MOG_{35\text{-}55}$-induced EAE. C57BL/6 mice were injected intraperitoneally with PBS or curdlan (5 μg/mouse). One day after treatment, mice were sensitized subcutaneously with the $MOG_{35\text{-}55}$ peptide emulsified in CFA. We found that all mice from the two groups had EAE; two out of ten EAE mice from the curdlan treatment group developed hyperacute EAE. The incidence of hyperacute EAE was significantly lower in $MOG_{35\text{-}55}$-induced EAE than in the $PLP_{139\text{-}151}$-induced EAE (73%, $P < 0.05$, χ^2 test).

Supplementary Table 2. Effects of curdlan in passive EAE.

**Mouse strain**	**SJL/J**		**C57BL/6**	
	Control	Curdlan	Control	Curdlan
Incidence (%)	7 / 8 (88%)	7 / 8 (88%)	2 / 8 (25%)	3 / 9 (33%)
Mortality (%)	0 / 8 (0%)	0 / 8 (0%)	0 / 8 (0%)	0 / 9 (0%)

Supplementary Table 2. We examined whether curdlan treatment could directly affect primed effector cells. Mononuclear cells (MNCs) were isolated from the spleen and inguinal lymph nodes of $PLP_{139\text{-}151}$-sensitized mice and re-stimulated with the $PLP_{139\text{-}151}$ peptide in the presence or absence of curdlan (1 μg/mL) for 3 days. To induce passive EAE, we injected the re-stimulated MNCs intravenously into irradiated SJL/J mice (4 Gy/mouse). We found that *in vitro* curdlan treatment neither altered the incidence of EAE nor induced hyperacute EAE. To further examine the effects of curdlan, we determined whether curdlan could alter the incidence of passive EAE following adoptive transfer of suboptimally activated $MOG_{35\text{-}55}$-specific T cells from naïve 2D2 mice, in which most T cells are $MOG_{35\text{-}55}$-specific and naïve/resting. For *in vitro* stimulation, we cultured MNCs from the spleen and inguinal lymph nodes of 2D2 mice with the $MOG_{35\text{-}55}$ peptide, anti-CD3 monoclonal antibody (mAb), anti-CD28 mAb, interleukin (IL)-6, IL-23, transforming growth factor (TGF)-β, anti-IFN-γ mAb, and anti-IL-4 mAb in the presence or absence of curdlan. After 3 days stimulation, we injected the MNCs intravenously into irradiated C57BL/6 mice (4 Gy/mouse). We found no significant differences in the incidence or mortality of EAE between the control and curdlan treatment groups. Thus, direct stimulation of MNCs with curdlan did not induce hyperacute EAE.
